# The Efficacy and Safety of Proton Pump Inhibitors Combining Dual Antiplatelet Therapy in Patients with Coronary Intervention: A Systematic Review, Meta-Analysis and Trial Sequential Analysis of Randomized Controlled Trials

**DOI:** 10.31083/j.rcm2408230

**Published:** 2023-08-09

**Authors:** Shichu Liang, Min Ma, Yonghao Chen, Jing Zhang, Jing Li, Shenglin Jiang, Yaoqun Wang, He Huang, Yong He

**Affiliations:** ^1^Department of Cardiology, West China Hospital, Sichuan University, 610041 Chengdu, Sichuan, China; ^2^Department of Cardiology, The Sixth People’s Hospital of Chengdu, 610072 Chengdu, Sichuan, China; ^3^Department of Gastroentrology, West China Hospital, Sichuan University, 610041 Chengdu, Sichuan, China; ^4^Research Center of Evidence-Based Medicine and Clinical Epidemiology, West China Hospital, Sichuan University, 610041 Chengdu, Sichuan, China; ^5^Division of Biliary Surgery, Department of General Surgery, West China Hospital, Sichuan University, 610041 Chengdu, Sichuan, China

**Keywords:** proton pump inhibitors, acute coronary syndrome, meta-analyses, dual antiplatelet therapy, sequential trial analysis

## Abstract

**Background:**

Proton pump inhibitors (PPIs) are used to prevent gastrointestinal hemorrhage in patients with coronary treatment undergoing dual antiplatelet therapy (DAPT).

**Methods:**

A systematic review was performed to compare the outcomes between DAPT and DAPT + PPI in acute coronary syndrome (ACS) patients or patients who took percutaneous coronary intervention (PCI) with coronary stent implantation (PCI patients), and to estimate, for the first time, the sample size needed for reliable results via trial sequential analysis (TSA). The PubMed, EMBASE, the Cochrane Library and Web of Science databases were searched for articles authored from the onset until November 1, 2022, for randomized controlled trials (RCTs) comparing outcomes in ACS or PCI patients who undertook DAPT or DAPT + PPI. The primary outcomes were the incidence rate of gastrointestinal events and major adverse cardiovascular events (MACEs).

**Results:**

The initial web search retrieved 786 literature references. Eventually, eight articles published between 2009 and 2020 were incorporated into the systematic review and meta-analysis. The combined results established a non-significant variation in MACEs incidences between the DAPT group and DAPT + PPI group [risk ratio (RR) = 0.93, 95% confidence interval (CI) = 0.81–1.06, *p* = 0.27, *$I^{2}$* = 0%]; conversely, the incidence of gastrointestinal events was significantly decreased in the DAPT + PPI group in comparison with the DAPT group (RR = 0.33, 95% CI = 0.24–0.45, *p <* 0.00001, *$I^{2}$* = 0%). TSA of MACEs and gastrointestinal events revealed that meta-analysis included adequate trials (required sample size = 6874) in the pool to achieve 80% study power.

**Conclusions:**

Based on our results, DAPT + PPI can significantly reduce gastrointestinal outcomes without affecting cardiovascular outcomes in PCI and ACS patients compared to DAPT.

## 1. Introduction

Globally, cardiovascular disorders are the main reason for mortality and disability, with coronary artery disease (CAD) being among the highest prevalent cardiovascular disorders, which may typically lead to acute myocardial infarction (AMI) and, ultimately, heart failure (HF) [1, 2]. Nowadays, with the unprecedented development of coronary revascularization, in particular, percutaneous coronary intervention (PCI), the prognosis of CAD patients, has been greatly improved [3]. Conventional dual antiplatelet therapy (DAPT) with aspirin and clopidogrel is a base for the treatment of antithrombosis following AMI and PCI; the recommended period of treatment is at least 12 months—the duration put forth in the 2019 recommendations from The European Society of Cardiology (ESC) [4]. Nevertheless, antithrombotic treatment not only decreases the incidence of ischemic incidents, but also elevates the probability of bleeding events, especially the incidence of gastrointestinal bleeding [5].

In the above-mentioned 2019 ESC guidelines, proton pump inhibitors (PPIs) are the first choice category recommendation when it comes to reducing gastrointestinal hemorrhage risk in patients medicated with DAPT and could be a successful therapy in terms of enhancing the safety and prognosis [4]. However, clopidogrel and PPIs share the same cytochrome enzyme cytochrome P450 2C19 (CYP2C19), and the drug-drug interactions have drawn widespread clinical attention [6]. It has been proven that PPIs can significantly decrease the inhibitory effect on the platelets of clopidogrel *in vitro* [7], which may result in thrombotic events such as myocardial infarction and revascularization.

In clinical trials, the results are conflicting and even contradictory in well-conducted observational research besides randomized controlled trials (RCTs) concerning the influence of PPIs on cardiovascular outcomes [8]. Some included observational trials lack data on PPI doses and may ascertain exposure [8, 9]. Thus, to provide more reasonable evidence for clinical practice, only RCTs were eligible for inclusion here. Moreover, a systematic review was carried out to compare the cardiovascular and gastrointestinal events between DAPT and DAPT + PPI in acute coronary syndrome (ACS) patients or patients with coronary stent (PCI patients), and to estimate, for the first time, the sample size needed to produce reliable results via trial sequential analysis (TSA).

## 2. Methods

### 2.1 Research Design and Literature Search

The present meta-analysis conformed to PRISMA (preferred reporting items for systematic reviews and meta-analyses) standards [10]. PROSPERO was used to register the protocol for this systematic review and meta-analysis (CRD42021289424). The Population, Intervention, Comparator, Outcome and Study design (PICOS) approach was used to frame the research objectives (Table 1). There were exclusions for non-human studies, conferences, reviews, case reports, and meta-analyses. Furthermore, investigations that did not evaluate the clinical results of DAPT + PPI versus DAPT in patients with ACS or PCI or those that used non-randomized administration of PPIs were excluded.

**Table 1.** **“PICOS” Method for choosing clinical trials in the systematic search**.

PICOS	
1 Participants	ACS patients or patients with coronary stent (PCI patients).
2 Intervention	The patients who took DAPT with PPI.
3 Comparison	The patients who took DAPT with placebo or without PPI.
4 Outcomes	The occurrence rate of major adverse cardiovascular events and gastrointestinal events.
5 Study design	Randomized controlled trials only.

ACS, acute coronary syndrome; DAPT, dual antiplatelet therapy; PCI, percutaneous coronary intervention; PPI, proton pump inhibitor; PICOS, The Population, Intervention, Comparator, Outcome and Study design.

The PubMed, EMBASE, the Cochrane Library and Web of Science databases were screened by two authors (SCL and YHC) separately for publication from initial to November 1, 2022, using the heading terms “dual antiplatelet therapy”, “DAPT”, “clopidogrel”, “P2Y12 receptor inhibitors”, “proton pump inhibitors (omeprazole, lansoprazole, esomeprazole, pantoprazole, rabeprazole)” and “PPI”. The scan was conducted by merging the subject and free terms. There were no language limitations. Also, the citations of related publications were scanned for additional eligible investigations.

The primary endpoints were major adverse cardiovascular events (MACEs) and gastrointestinal incidents. MACEs are characterized by composite cardiovascular events, including angina pectoris, secondary heart failure, severe arrhythmia, cardiac death, recurrent myocardial infarction, revascularization, in-stent thrombosis, ischemic stroke, as well as a transient ischemic attack (TIA). The gastrointestinal events include gastrointestinal bleeding (such as overt gastroduodenal hemorrhage, overt upper gastrointestinal hemorrhage of unknown origin, occult bleeding), gastrointestinal ulcers (such as gastrointestinal pain with underlying multiple erosive diseases and symptomatic gastroduodenal ulcer), and gastroesophageal reflux disease. The secondary cardiovascular endpoints were cardiac death, all-cause death, recurrent myocardial infarction, revascularization, in-stent thrombosis, ischaemic stroke, and TIA. The secondary gastrointestinal endpoints were gastrointestinal ulcers and gastrointestinal bleeding (including upper gastrointestinal bleeding).

### 2.2 Data Collection and Quality Evaluation

The same researchers (SCL and YHC) who completed the literature search and study selection also extracted the data. They were not blinded to the study authors and organizations. Contradictions were resolved by a third viewer (MM). Moreover, HH and YH oversaw the entire procedure. Two authors separately extracted these data: the first author, year of publishing, sample size and demographic characters in the DAPT and DAPT + PPI groups, the follow-up time, and the incidence of outcomes of efficacy and safety.

The Cochrane Handbook of Systematic Reviews and a revised Jadad quality scale were employed for the quality evaluation [11, 12]. A Jadad score from 4 to 7 indicates good quality. Using Stata v15.0 (The StataCorp LP, College Station, TX, US), publication bias was evaluated utilizing funnel plots. GRADE (Grading Recommendations Assessment, Development, and Evaluation) was utilized to examine the entire confidence of evidence for every outcome [13]. The summarization of results table was developed using the GRADEpro Guideline Development Tool (https://www.gradepro.org).

### 2.3 Statistical Analysis and Meta-Analysis 

All data were analyzed appropriately utilizing RevMan v5.3.5 (The Cochrane Collaboration, Copenhagen, Denmark). PRISMA compiled the final results. The two authors who collected the data (SCL and YHC) were not blinded to the research authors and organizations. Statistical heterogeneity was conducted via the I-square test. Heterogeneity was determined to be absent (*$I^{2}$*: 0%–25%), low (*$I^{2}$*: 25.1%–50%), moderate (*$I^{2}$*: 50.1%–75%), or high (*$I^{2}$*: 75.1%–100%). When the quantity of research was relatively limited, the employment of a random-effects model was examined, which predicted the continuous outcome results if the *p* was 0.1. The *$I^{2}$* was >50% demonstrates statistical heterogeneity [14]. Other than that, a fixed-effects model was utilized. A *p* < 0.05 was seen as indicating statistical significance.

### 2.4 Trial Sequential Analysis

Spurious findings can be caused by random errors when a meta-analysis comprises a limited quantity of trials and patients [15], and in such a situation, a TSA is conducted. The index is set following the guideline: (a) Conventional Test Boundary: boundary type: two-sided, type 1 error: 5%; (b) Alpha-spending boundary: type 1 error: 5%, power: 80%, relative risk reduction (RRR): 35%, Incidence in control arm: 3%; (c) Law of the Iterated Logarithm: type 1 error: 5%, penalty λ: 1.5 [16]. The TSA was conducted via Trial Sequential Analysis v.0.9.5.10 beta program (Copenhagen Trial Unit, Centre for Clinical Intervention Research, Rigshospitalet, Copenhagen, Denmark, https://www.ctu.dk/tsa).

## 3. Results

### 3.1 Strategy and Selection of Study

**Fig. 1.**
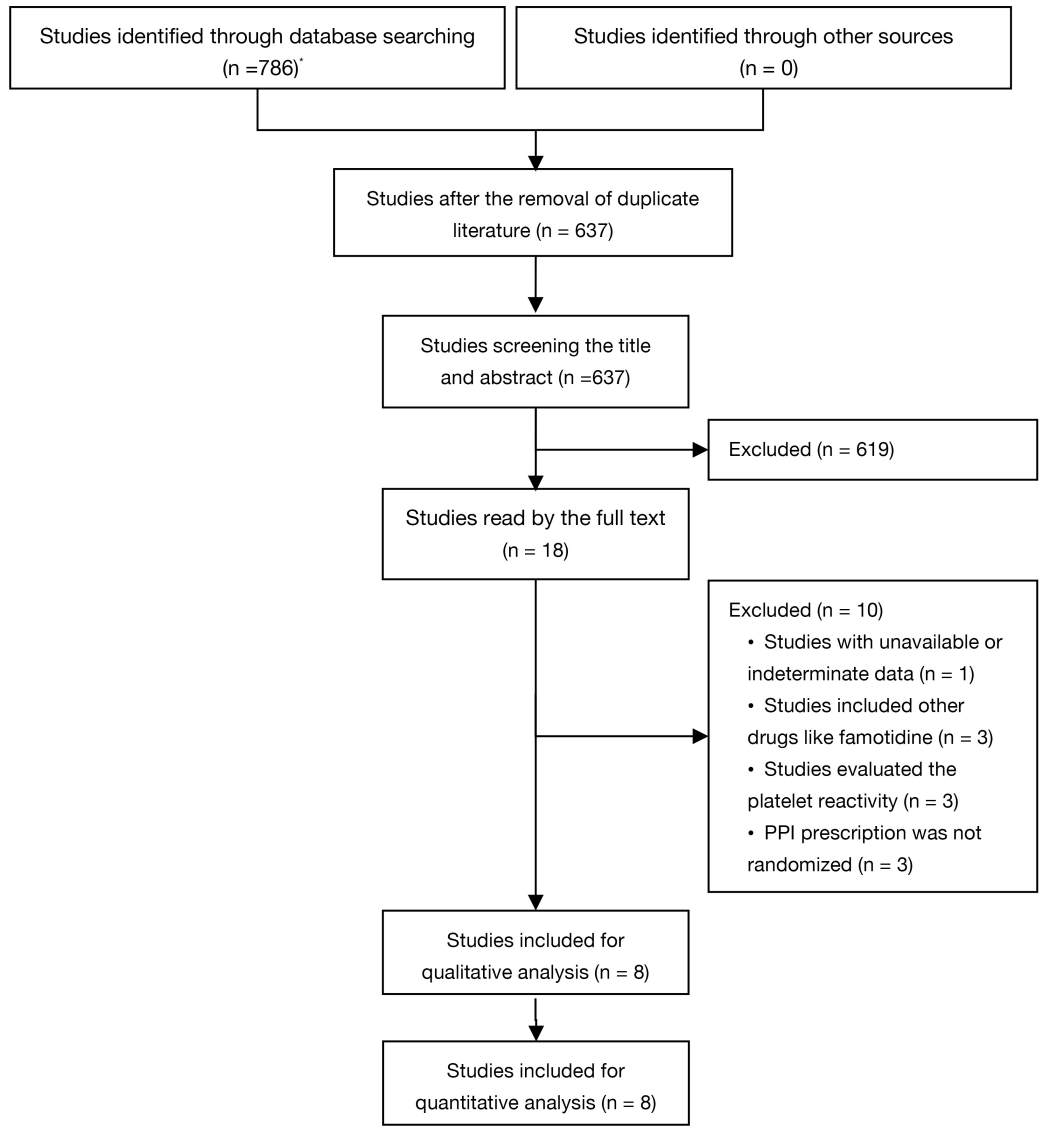
Flow chart of the process (*247 from PubMed, 87 from EMBASE, 234 from The Cochrane Library, and 218 from Web of Science).

The online search initially yielded 786 literature citations (247 from PubMed, 87 from EMBASE, 234 from The Cochrane Library, and 218 from Web of Science). Following the deletion of 149 duplicates, 637 literature items remained, and 619 were excluded after a review of the titles and keywords because of non-relevance or repetition. Two authors (SCL and YHC) evaluated 18 abstracts and chose ten articles for full-text examination. In total, ten studies were excluded due to unavailable or indeterminate data (n = 1), including famotidine (n = 3), evaluating the platelet reactivity (n = 3), and the PPI prescription was not randomized (n = 3). The search strategy and excluded studies can be seen in the **Supplementary Materials**. Fig. 1 displays the PRISMA flowchart illustrating the systematic literature search and research selection criteria.

### 3.2 Data Extraction and Quality Assessment

Eventually, eight studies [17, 18, 19, 20, 21, 22, 23, 24, 25] published from 2009 to 2020 were included in the meta-analysis. Table 2 (Ref. [17, 18, 19, 20, 21, 22, 23, 24, 25]) shows the details of the studies. Of those, seven investigations utilized aspirin + clopidogrel as DAPT protocol [17, 18, 19, 20, 21, 22, 23, 24], one study [25] utilized aspirin + ticagrelor as DAPT protocol, and one study [24] used aspirin + clopidogrel/ticagrelor as DAPT protocol. Among these studies, five of them [17, 18, 19, 22, 25] used omeprazole as the PPI, while three studies [20, 21, 24] employed pantoprazole as the PPI. The quality assessment demonstrated an acceptable overall risk of bias and applicability concerns in most articles, although one study [21] had low Jadad scores.

**Table 2.** **Basic information of included studies**.

Study	Country	Study population	Intervention		DAPT type	PPI type	Number (T/C)	Age (T/C)	Male (T/C)	Mean follow-up time	Endpoints	Jadad score
			T	C								
Gao 2009 [17]	China	ACS patients	DAPT + PPI	DAPT + Placebo	Aspirin + Clopidogrel	Omeprazole	114/123	58.2 ± 8.7/	126/111	14 days	(3)(5)(8)(10)(11)	5
								57.5 ± 9.2				
Bhatt 2010 [18]	Spain and USA	ACS patients or PCI patients	DAPT + PPI	DAPT + Placebo	Aspirin + Clopidogrel	Omeprazole	1876/1885	68.5 (60.7–74.4)/	1255/1308	106 days	(1)(2)(3)(5)(7)(8)(9)(10)	6
								68.7 (60.6–74.7)				
Ren 2011 [19]	China	ACS patients	DAPT + PPI	DAPT + Placebo	Aspirin + Clopidogrel	Omeprazole	86/86	62.08 ± 10.62/	62/63	30 days	(4)(7)(8)(10)(11)	4
								61.84 ± 11.21				
Wu 2011 [20]	China	ACS patients	DAPT + PPI	DAPT + Placebo	Aspirin + Clopidogrel	Pantoprazole	333/332	NR	246/244	30 days	(3)(8)(10)	4
Wei 2016 [21]	China	ACS patients	DAPT + PPI	DAPT	Aspirin + Clopidogrel	Pantoprazole	123/84	59.32 ± 9.14/	69/48	6 months	(1)(2)(4)(8)(10)	3
								58.47 ± 10.06				
Vaduganathan 2016 [22, 23]	Spain and USA	ACS patients or PCI patients	DAPT + PPI	DAPT + Placebo	Aspirin + Clopidogrel	Omeprazole	1869/1883	68.2 ± 10.2; 63.6 ± 1.4/	1249/1307	110 days	(1)(2)(3)(4)(6)(7)(8)(9)(10)(11)	7
								68.0 ± 10.4; 63.8± 11.3				
Jensen 2017 [24]	Denmark	PCI patients	DAPT + PPI	DAPT	Aspirin + Clopidogrel/	Pantoprazole	997/1012	64.7 ± 10.2/	729/758	1 year	(1)(3)(4)(8)(9)(10)(11)	5
					Ticagrelor			64.8 ± 10.6				
Zhang 2020 [25]	China	ACS patients	DAPT + PPI	DAPT	Aspirin + Ticagrelor	Omeprazole	43/43	60.2 ± 3.6/	31/29	6 months	(1)(8)(10)	4
								59.5 ± 3.5				

ACS, acute coronary syndrome; DAPT, dual antiplatelet therapy; PCI, percutaneous coronary intervention; PPI, proton pump inhibitor; NR, not reported; T, experimental group; C, control group. Endpoints: (1) Major adverse cardiovascular event; (2) Cardiac death; (3) All-cause death; (4) Recurrent myocardial infarction; (5) Revasculation; (6) In-stent restenosis; (7) Stroke; (8) Gastrointestinal events; (9) Gastrointestinal ulcers; (10) Gastrointestinal bleeding; (11) Upper gastrointestinal bleeding.

### 3.3 Cardiovascular and Gastrointestinal Outcomes

**Fig. 2.**
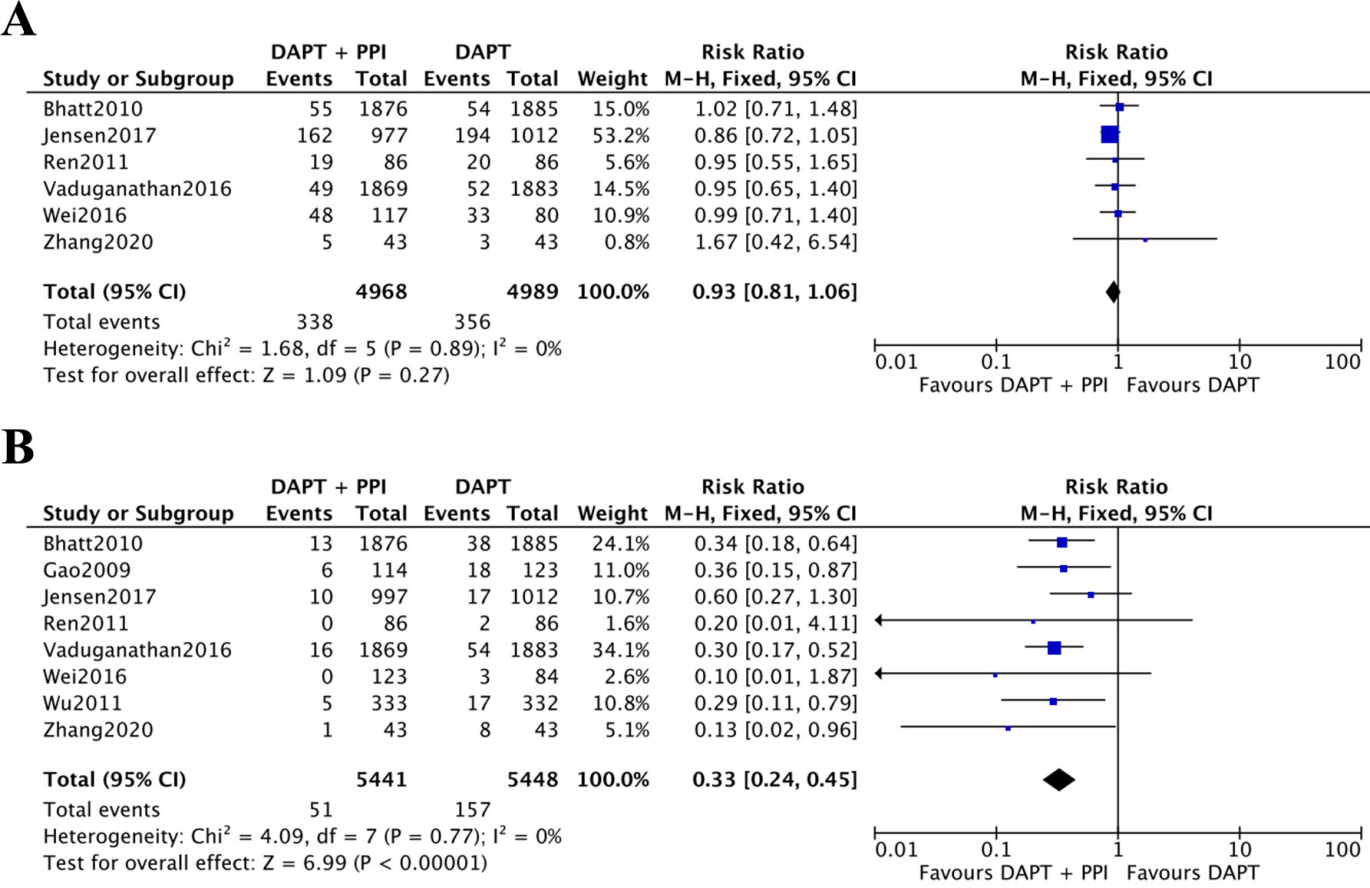
**The results of meta-analysis**. (A) Incidence of MACEs between DAPT + PPI and DAPT groups. (B) Incidence of gastrointestinal events between the DAPT + PPI and the DAPT groups. CI, confidence interval; DAPT, dual antiplatelet therapy; PPI, proton pump inhibitor; MACEs, major adverse cardiovascular events.

In total, six studies [18, 19, 21, 22, 24, 25] reported the incidence of MACEs (Fig. 2A). Non-significant variation was observed in the instances of MACEs between the two groups, with 4968 and 4989 patients in the DAPT + PPI and DAPT groups, respectively (RR = 0.93, 95% CI = 0.81–1.06, *p* = 0.27, *$I^{2}$* = 0%). Moreover, eight studies [17, 18, 19, 20, 21, 22, 23, 24, 25] reported the incidence of gastrointestinal events (Fig. 2B). The occurrence of these events was reduced significantly in the DAPT + PPI group compared to patients in the DAPT controls (RR = 0.33, 95% CI = 0.24–0.45, *p <* 0.00001, *$I^{2}$* = 0%). Table 3 (Ref. [17, 18, 19, 20, 21, 22, 23, 24, 25]) illustrates subgroup analysis results of MACEs and gastrointestinal events. **Supplementary Table 1** shows the secondary endpoint results. Table 4 summarizes the results for all findings involving evidence certainty.

**Table 3.** **Findings subgroup analysis of MACEs and gastrointestinal events**.

MACEs	Studies	Heterogeneity		Effects model	Meta analsysis		GI events	Studies	Heterogeneity		Effects model	Meta analsysis	
		*p* value	* $I^{2}$ *		Effect index (95% CI)	*p* value			*p* value	* $I^{2}$ *		Effect index (95% CI)	*p* value
Type of PPIs													
Omeprazole	4 [18, 19, 22, 23, 25]	0.88	0%	Fixed	RR 1.00 (0.79–1.26)	0.98	Omeprazole	5 [17, 18, 19, 22, 23, 25]	0.90	0%	Fixed	RR 0.31 (0.21–0.44)	<0.00001
Pantoprazole	2 [21, 24]	0.48	0%	Fixed	RR 0.89 (0.75–1.05)	0.16	Pantoprazole	3 [20, 21, 24]	0.31	11%	Fixed	RR 0.41 (0.23–0.73)	0.002
Type of DAPT													
Aspirin + Clopidogrel	4 [18, 19, 21, 22, 23]	0.99	0%	Fixed	RR 0.98 (0.81–1.20)	0.88	Aspirin + Clopidogrel	6 [17, 18, 19, 20, 21, 22, 23]	0.97	0%	Fixed	RR 0.31 (0.22–0.44)	<0.00001
Aspirin + Ticagrelor	1 [25]	-	-	-	RR 1.67 (0.42–6.54)	0.73	Aspirin + Ticagrelor	1 [25]	-	-	-	RR 0.13 (0.02–0.96)	0.05
Follow-up time													
>6 months	2 [21, 25]	0.47	0%	Fixed	RR 1.04 (0.75–1.45)	0.81	>6 months	2 [21, 25]	0.89	0%	Fixed	RR 0.12 (0.02–0.62)	0.01
<6 months	4 [18, 19, 22, 23, 24]	0.86	0%	Fixed	RR 0.91 (0.78–1.06)	0.22	<6 months	6 [17, 18, 19, 20, 22, 23, 24]	0.79	0%	Fixed	RR 0.35 (0.26–0.48)	<0.00001

CI, confidence interval; DAPT, dual antiplatelet therapy; GI, gastrointestinal; MACEs, major adverse cardiovascular events; PPIs, proton pump inhibitors; RR, risk ratio.

**Table 4.** **GRADE summary of the findings**.

Certainty assessment							№ of patients		Effect		Certainty	Importance
№ of studies	Study design	Risk of bias	Inconsistency	Indirectness	Imprecision	Other considerations	DAPT + PPI	DAPT	Relative	Absolute		
									(95% CI)	(95% CI)		
Major adverse cardiovascular events												
6	Randomised trials	${\mathrm{Serious}}^{a}$	Not serious	${\mathrm{Serious}}^{b}$	Not serious	${\mathrm{Publication bias strongly suspected}}^{c}$	338/4968 (6.8%)	356/4989 (7.1%)	RR 0.93	5 fewer per 1000	⊕◯◯◯	CRITICAL
									(0.81 to 1.06)	(from 14 fewer to 4 more)	Very low	
Cardiac death												
3	Randomised trials	${\mathrm{Serious}}^{d}$	${\mathrm{Serious}}^{e}$	Not serious	Not serious	${\mathrm{Publication bias strongly suspected}}^{c}$	13/3862 (0.3%)	8/3848 (0.2%)	RR 1.49	1 more per 1000	⊕◯◯◯	CRITICAL
									(0.62 to 3.57)	(from 1 fewer to 5 more)	Very low	
All-cause death												
5	Randomised trials	Not serious	Not serious	Not serious	Not serious	None	59/5189 (1.1%)	81/5235 (1.5%)	RR 0.74	4 fewer per 1000	⊕ ⊕ ⊕ ⊕	CRITICAL
									(0.53 to 1.02)	(from 7 fewer to 0 fewer)	High	
Recurrent myocardial infarction												
5	Randomised trials	Not serious	Not serious	Not serious	Not serious	None	168/4902 (3.4%)	178/4853 (3.7%)	RR 0.94	2 fewer per 1000	⊕ ⊕ ⊕ ⊕	CRITICAL
									(0.77 to 1.15)	(from 8 fewer to 6 more)	High	
Revascularization												
3	Randomised trials	Not serious	Not serious	Not serious	Not serious	None	90/3859 (2.3%)	99/3891 (2.5%)	RR 0.92	2 fewer per 1000	⊕ ⊕ ⊕ ⊕	CRITICAL
									(0.70 to 1.22)	(from 8 fewer to 6 more)	High	
In-Stent thrombosis												
1	Randomised trials	Not serious	${\mathrm{Serious}}^{e}$	Not serious	Not serious	${\mathrm{Publication bias strongly suspected}}^{c}$	0/43 (0.0%)	2/43 (4.7%)	RR 0.20	37 fewer per 1000	⊕⊕◯◯	CRITICAL
									(0.01 to 4.05)	(from 46 fewer to 142 more)	Low	
Ischaemic stroke and transient ischaemic attack												
4	Randomised trials	Not serious	${\mathrm{Serious}}^{e}$	Not serious	Not serious	None	9/3874 (0.2%)	6/3897 (0.2%)	RR 1.47	1 more per 1000	⊕⊕⊕◯	CRITICAL
									(0.54 to 3.97)	(from 1 fewer to 5 more)	Moderate	
Gastrointestinal events												
8	Randomised trials	Not serious	Not serious	${\mathrm{Serious}}^{f}$	Not serious	None	51/5441 (0.9%)	157/5448 (2.9%)	RR 0.33	19 fewer per 1000	⊕⊕⊕◯	CRITICAL
									(0.24 to 0.45)	(from 22 fewer to 16 fewer)	Moderate	
Gastrointestinal ulcer												
3	Randomised trials	Not serious	Not serious	Not serious	Not serious	None	11/4742 (0.2%)	31/4780 (0.6%)	RR 0.36	4 fewer per 1000	⊕ ⊕ ⊕ ⊕	CRITICAL
									(0.18 to 0.71)	(from 5 fewer to 2 fewer)	High	
Gastrointestinal bleeding												
8	Randomised trials	${\mathrm{Serious}}^{g}$	Not serious	${\mathrm{Serious}}^{h}$	Not serious	None	40/5441 (0.7%)	128/5448 (2.3%)	RR 0.31	16 fewer per 1000	⊕⊕◯◯	CRITICAL
									(0.22 to 0.44)	(from 18 fewer to 13 fewer)	Low	
Upper gastrointestinal Bleeding												
4	Randomised trials	Not serious	Not serious	Not serious	Not serious	None	16/2308 (0.7%)	47/2397 (2.0%)	OR 0.35	13 fewer per 1000	⊕ ⊕ ⊕ ⊕	CRITICAL
									(0.20 to 0.62)	(from 16 fewer to 7 fewer)	High	

CI, confidence interval; DAPT, dual antiplatelet therapy; PPI, proton pump inhibitor; RR, risk ratio. ⊕⊕⊕⊕, high quality; ⊕⊕⊕◯, moderate quality; ⊕⊕◯◯, low quality; ⊕◯◯◯, very low quality. Explanations: ^a^. 1 study (Wei 2016) has low quality, and its involvement value in this result is 10.9%, which decreases our certainty of effect. ^b^. The definition of major adverse cardiovascular events (MACEs) varies among studies. ^c^. Strongly suspected publication bias lowers our certainty in effect. ^d^. 1 study (Wei2016) has low quality, and its involvement value in this result is 28.4%, which decreases our certainty of effect. ^e^. Substantial confidence intervals do not eliminate significant advantage or damage, which reduces our certainty of effect. ^f^. The definition of gastrointestinal events varies from studies. ^g^. 1 study (Wei 2016) has low quality; its involvement value in this result is 11.9%, which reduces our certainty in effect. ^h^. The definition of gastrointestinal bleeding varies among studies.

### 3.4 Trial Sequential Analysis

**Fig. 3.**
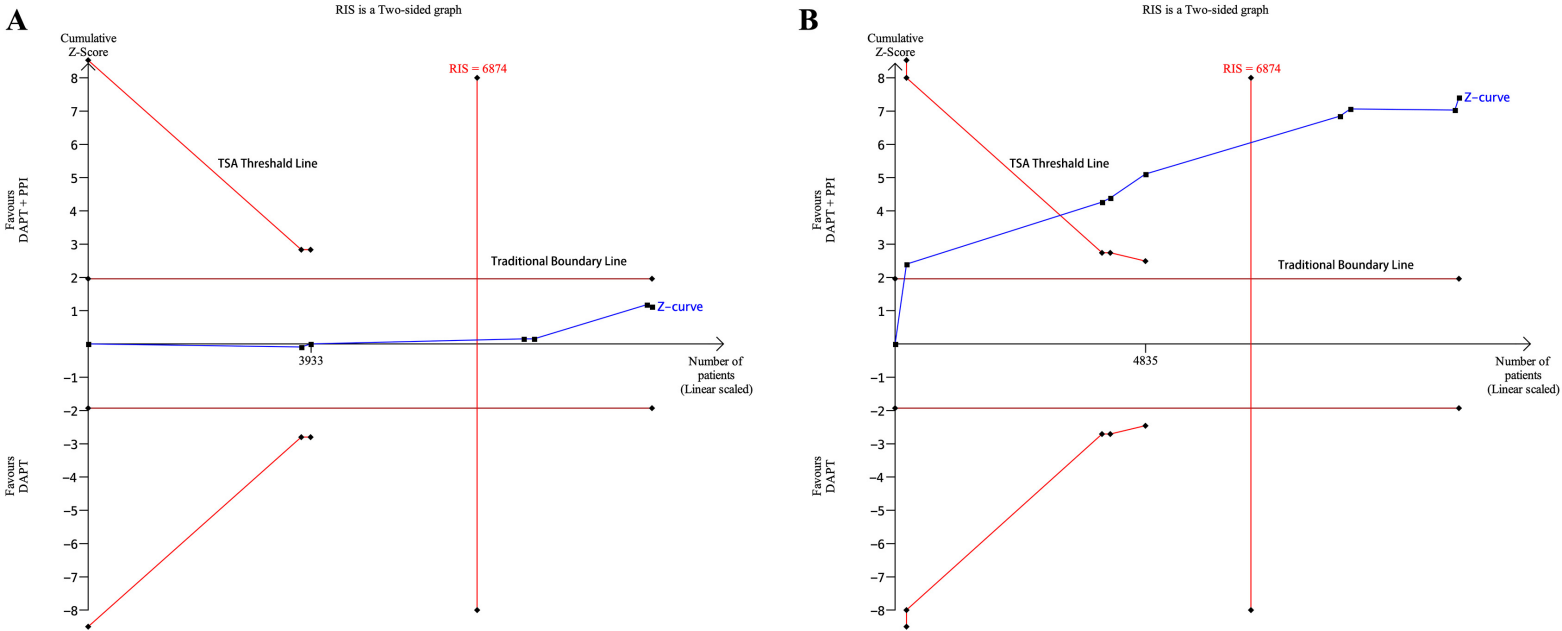
**The results of trial sequential analysis**. (A) TSA of MACEs between the DAPT + PPI and DAPT groups. (B) TSA of gastrointestinal events between the DAPT + PPI and DAPT groups. CI, confidence interval; DAPT, dual antiplatelet therapy; PPI, proton pump inhibitor; RIS, recommended information size; TSA, trial sequential analysis; MACEs, major adverse cardiovascular events.

TSA of MACEs demonstrated that, although the cumulative Z-value curve did not cross either the traditional boundary value or the TSA threshold line, the total sample size exceeded the recommended information size (RIS, sample size = 9957, RIS = 6874), indicating that no statistical difference could be highlighted between the two groups (Fig. 3A), and no more studies are needed. The TSA of gastrointestinal events depicted that the cumulative Z-value curve crossed both the traditional boundary value and the TSA threshold line, and the RIS was achieved (sample size = 10,889, RIS = 6874), and no further research is required (Fig. 3B).

## 4. Discussion

This study detected eight RCTs with 5441 patients medicated with DAPT + PPI and 5448 patients medicated with DAPT only or DAPT + placebo. The results demonstrate that DAPT + PPI probably has no significant impact on cardiovascular outcomes such as MACEs in patients with coronary intervention, while a specific decrease was displayed in gastrointestinal events, such as gastrointestinal ulcers and gastrointestinal bleeding (including upper gastrointestinal bleeding). To the best of our knowledge, this is the first study to conduct a TSA, and the results provided firm evidence regarding the benefit of cardiovascular and gastrointestinal outcomes associated with DAPT + PPI.

DAPT in ACS patients subjected to coronary stent implantation for at least 6 to 12 months is the IA recommendation [26, 27], but it must be noted that gastrointestinal bleeding can be caused by DAPT. PPIs are indicated for patients who suffer from a higher-than-average probability of gastrointestinal hemorrhage to decrease gastrointestinal outcomes [26, 27]. The metabolism of clopidogrel may be affected by PPIs, as they share the same metabolizing enzymes: CYP2C19. Gilard* et al*. [7] first observed that the PPI treatment might diminish the biological action of clopidogrel *in vitro* and then revealed that omeprazole can significantly decrease the action of clopidogrel on inhibiting platelet P2Y12 in an RCT [28]. Concerns were raised, as the low bioactivities of clopidogrel might result in ischemic events. Subsequent experiments [29, 30, 31] revealed that pantoprazole, esomeprazole, and rabeprazole do not influence the antiplatelet effect of clopidogrel, thus suggesting that they are more suitable for the combination of DAPT. However, in real-world studies, researchers found that the cutoff of clinically significant poor response to clopidogrel is fairly higher than that commonly achieved by PPI treatment [32]. The cardiovascular outcomes among the two groups are insignificant. This result has been confirmed by our study and previous studies [9, 19, 21].

Ticagrelor, a novel, oral, direct-acting P2Y12 inhibitor, does not need to be metabolized via CYP2C19, thus meaning that its inhibitory effects are not impacted by PPIs [33]. The PLATO trial first illustrated that, compared to clopidogrel, ticagrelor could significantly reduce the rate of MACEs (9.8% vs. 11.7%, *p* < 0.001) with no rise in the total frequency of severe hemorrhage (11.6% vs. 11.2%, *p* = 0.43) [34]. However, the incidence rate of gastrointestinal bleeding was significantly increased (1.3% vs. 1.0%, *p* = 0.048) [35]. The GLOBAL LEADERS trial also demonstrated that the combination of PPI and ticagrelor monotherapy might be safe. Nevertheless, the utilization of PPIs was not randomized, and the unknown confounding factors should be considered [36]. In our study, only one included RCT with 86 patients compared the combination of PPI with aspirin and ticagrelor. They found a non-significant variation in MACEs incidence rate, while the incidence rate of gastrointestinal bleeding significantly declined [25]. For patients with high bleeding risk, de-escalation from ticagrelor to clopidogrel is common [37]. There is still an absence of adequate proof regarding the combination of PPI with aspirin and ticagrelor, and more studies are needed in the future [38].

It should be noted that although PPIs are used to reduce gastrointestinal outcomes such as gastrointestinal ulcers and upper gastrointestinal bleeding in high-risk patients [39], lower gastrointestinal complications might arise due to PPI use [40]. The first three months is the high-risk period for both upper and lower gastrointestinal hemorrhage in PCI patients undergoing DAPT, and the incidence of lower gastrointestinal hemorrhage is higher than that of upper gastrointestinal bleeding [41]. According to researchers, short-term (six months) DAPT followed by P2Y12 inhibitor monotherapy can lower the incidence of severe hemorrhage after PCI without elevating the risk of AMI [42]. In addition, the OPTION trial depicted that indobufen + clopidogrel DAPT, compared to aspirin + clopidogrel DAPT, significantly decreased gastrointestinal bleeding, thus meaning that the former may be a safer choice in the future [43]. Nevertheless, the OPT-PEACE study demonstrated that almost every patient who received single antiplatelet therapy (SAPT) or DAPT experienced a gastrointestinal injury; however, hemorrhage was uncommon [44]. SAPT and DAPT cause injuries in the upper and lower digestive tract. Washio* et al*. [45] indicated that PPIs raised the probability of short-term nonsteroidal anti-inflammatory drug-induced minor intestinal damage, possibly due to the altered luminal environment caused by the substantial inhibition of stomach acid secretion [45, 46]. The small-intestinal mucosal damage may be exacerbated by the altered microbiota [47].

There are, as yet, no effective preventive measures for lower gastrointestinal bleeding. During the use of DAPT, attention should be paid to monitoring patients’ symptoms, their fecal occult blood test results, and their blood routine. Therefore, although PPIs effectively reduce upper gastrointestinal complications, lower gastrointestinal complications might rise due to PPI use [40]. Taking these confounding factors into consideration, the true effect of PPIs on the whole DAPT-related gastrointestinal bleeding needs to be further verified with more RCTs [40]. Future studies can distinguish between lower and upper gastrointestinal bleeding via magnetically controlled capsule endoscopy and other new technologies.

The strengths of our research include a pre-registered process, a TSA for estimating sample size, and a GRADE assessment of the certainty of evidence. Nevertheless, it has certain drawbacks. First, the insufficient granularity regarding the types of DAPT (i.e., ticagrelor), the types of patients (i.e., patients at high risk of experiencing thrombosis and hemorrhage), and the types of PPIs (i.e., lansoprazole, esomeprazole, and rabeprazole) may affect risk adjustment. What is more, the incorporated investigations were heterogenous in some results regarding the various definitions of MACEs, gastrointestinal events, and gastrointestinal bleeding. Fortunately, the clinical heterogeneity was not reflected in statistically significant discrepancy among any of the desired results. Despite our efforts to restrict the analysis to studies that involved patients taking aspirin and clopidogrel or ticagrelor, one study [24] also enrolled patients who took prasugrel. However, even if included, these patients accounted for only 0.02% of the sample and would thus not be likely to critically affect the results. Though the TSA showed that the meta-analysis pool had sufficient studies (RIS = 6874) to reach 80% study power, we think more large-scale RCTs with other types of PPIs are still needed in the future to explore its effects on lower gastrointestinal bleeding.

## 5. Conclusions

In patients with coronary intervention, compared to DAPT, DAPT + PPI can significantly reduce gastrointestinal outcomes without affecting cardiovascular outcomes. DAPT + PPI has a significant protective effect on gastrointestinal ulcers and upper gastrointestinal bleeding, while to determine its protective impact on lower gastrointestinal bleeding, further large-scale studies are required.

## Data Availability

All data generated or analyzed during this study are included in this published article.

## Supplementary Material

Supplementary material associated with this article can be found, in the online version, at https://doi.org/10.31083/j.rcm2408230.
