# Supplementary material for: The Efficacy and Safety of Proton Pump Inhibitors Combining Dual Antiplatelet Therapy in Patients with Coronary Intervention: A Systematic Review, Meta-Analysis and Trial Sequential Analysis of Randomized Controlled Trials
Source: Rev Cardiovasc Med. 2023 Aug 9;24(8):230. doi: 10.31083/j.rcm2408230 (PMC11266793; doi:10.31083/j.rcm2408230)
Supplement: Supplementary file 1 [file 2153-8174-24-8-230-s1.zip › 2153-8174-24-8-230-s1/Supplementary Materials.docx]

**Supplementary Materials**

**Search strategy**

**1.Pubmed (247)**

#1 proton pump inhibitors [MeSH Terms] OR proton pump inhibitors [Title/Abstract] OR PPI [Title/Abstract] OR omeprazole [Title/Abstract] OR lansoprazole [Title/Abstract] OR esomeprazole [Title/Abstract] OR pantoprazole [Title/Abstract]

#2 dual anti-platelet therapy [MeSH Terms] OR dual anti-platelet therapy [Title/Abstract] OR dual anti platelet therapy [Title/Abstract]

#3 coronary artery disease [MeSH Terms] OR coronary artery disease [Title/Abstract] OR CAD [Title/Abstract]

#4 acute coronary syndrome [MeSH Terms] OR acute coronary syndromes [Title/Abstract] OR ACS [Title/Abstract]

#5 percutaneous coronary intervention [MeSH Terms] OR percutaneous coronary intervention [Title/Abstract] OR PCI [Title/Abstract]

#6 #3 OR #4 OR #5

#7 randomized controlled trial [Publication Type] OR randomized controlled trials [MeSH Terms]

#8 randomized controlled trial [Title/Abstract] OR randomized controlled trials [Title/Abstract] OR RCT [Title/Abstract] OR RCTs [Title/Abstract] OR randomized controlled trials [Title/Abstract] OR randomized controlled trial [Title/Abstract] OR randomized clinical trial [Title/Abstract] OR randomized clinical trials [Title/Abstract])

#9 #7 OR #8

#10 #1 AND #6

#11 #2 AND #6

#12 #10 OR #11

#13 #12 AND #9

**2.Embase (87)**

#1 'proton pump inhibitors':ab,ti OR 'PPI':ab,ti OR 'omeprazole':ab,ti OR 'lansoprazole':ab,ti OR 'esomeprazole':ab,ti OR 'pantoprazole':ab,ti

#2 'dual anti-platelet therapy':ab,ti OR 'dual anti platelet therapy':ab,ti

#3 'coronary artery disease':ab,ti OR 'CAD':ab,ti OR 'acute coronary syndrome':ab,ti OR 'ACS':ab,ti OR 'percutaneous coronary intervention':ab,ti OR 'PCI':ab,ti

#4 'randomized controlled trial':ab,ti OR 'randomized controlled trials':ab,ti OR 'RCT':ab,ti OR 'RCTs':ab,ti OR 'randomized controlled trials':ab,ti OR 'randomized controlled trial':ab,ti OR 'randomized clinical trial':ab,ti OR 'randomized clinical trials':ab,ti

#5 #1 AND #3

#6 #2 AND #3

#7 #5 OR #6

#8 #7 AND #4

1. **The Cochrane Library (234)**

#1 MeSH descriptor:[Proton Pump Inhibitors] explode all trees (1570)

#2 (proton pump inhibitors):ti,ab,kw OR (PPI):ti,ab,kw OR (omeprazole):ti,ab,kw OR (lansoprazole):ti,ab,kw OR (esomeprazole):ti,ab,kw (9674)

#3 (pantoprazole):ti,ab,kw (1325)

#4 #1 OR #2 OR #3 (10298)

#5 MeSH descriptor: [Dual Anti-Platelet Therapy] explode all trees (73)

#6 (dual anti-platelet therapy):ti,ab,kw OR (dual anti platelet therapy):ti,ab,kw (336)

#7 #5 OR #6 (337)

#8 MeSH descriptor: (Coronary Artery Disease) explode all trees (7254)

#9 (coronary artery disease): ti,ab,kw OR (CAD):ti,ab,kw (26794)

#10 MeSH descriptor: (Acute Coronary Syndrome) explode all trees (2328)

#11 (acute coronary syndrome):ti,ab,kw OR (ACS):ti,ab,kw (6285)

#12 MeSH descriptor:[Percutaneous Coronary Intervention] explode all trees (6138)

#13 (percutaneous coronary intervention):ti,ab,kw OR (PCI):ti,ab,kw (14506)

#14 #8 OR #9 OR #10 OR #11 OR #12 OR #13 (41546)

#15 MeSH descriptor: [Randomized Controlled Trial] explode all trees (118)

#16 (randomized controlled trial):pt OR (randomized controlled trial)ti,ab,kw OR (randomized controlled trial):ti,ab,kw OR (RCT):ti,ab,kw OR (RCTs):ti,ab,kw (688558)

#17 (randomized controlled trials):ti,ab,kw OR (randomized controlled trial):ti,ab,kw OR (randomized clinical trial):ti,ab,kw OR (randomized clinical trials):ti,ab,kw (755279)

#18 #15 OR #16 OR #17 (761438)

#19 #4 AND #14 (190)

#20 #7 AND #14 (243)

#21 #19 OR #20 (421)

#22 #21 AND #18 (234)

**4. Web of Science (218)**

#1 (((((TS=(proton pump inhibitors)) OR TS=(PPI)) OR TS=(omeprazole)) OR TS=(lansoprazole)) OR TS=(esomeprazole)) OR TS=(pantoprazole)

#2 (TS=(dual anti-platelet therapy)) OR TS=(dual anti platelet therapy)

#3 (TS=(coronary artery disease)) OR TS=(CAD)

#4 (TS=(acute coronary syndromes)) OR TS=(ACS)

#5 (TS=(percutaneous coronary intervention)) OR TS=(PCI)

#6 #3 OR #4 OR #5

#7 (((((((TS=(randomized controlled trial)) OR TS=(randomized controlled trials)) OR TS=(RCT)) OR TS=(RCTs)) OR TS=(randomized controlled trials)) OR TS=(randomized controlled trial) OR TS=(randomized clinical trial)) OR TS=(randomized clinical trials))

#8 #1 AND #6

#9 #2 AND #6

#10 #8 OR #9

#11 #10 AND #7

**Supplementary Table 1 The results of meta-analysis of other cardiovascular and gastrointestinal outcomes.**

| **Outcome** | **Studies** | **Heterogeneity** | | **Effects model** | **Meta-analysis** | |
| --- | --- | --- | --- | --- | --- | --- |
|  |  | ***P* value** | ***I^2^*** |  | **Effect index (95% CI)** | ***P* value** |
| **Cardiovascular Outcomes** | | | | | | |
| **Cardiac Death** | 3 [18,21-23] | 0.89 | 0% | Fixed | RR 1.49 (0.62-3.57) | 0.37 |
| **All-cause Death** | 5 [17,18,20,22-24] | 0.13 | 44% | Fixed | RR 0.74 (0.53-1.02) | 0.07 |
| **Recurrent Myocardial Infarction** | 5 [18,21-25] | 0.96 | 0% | Fixed | RR 0.94 (0.77-1.15) | 0.57 |
| **Revascularization** | 3 [17,18,22-23] | 0.77 | 0% | Fixed | RR 0.92 (0.70-1.22) | 0.57 |
| **In-Stent Thrombosis** | 1 [25] | - | - | - | RR 0.20 (0.01-4.05) | 0.29 |
| **Ischaemic Stroke and Transient Ischaemic Attack** | 4 [18-19,22-23,25] | 0.79 | 0% | Fixed | RR 1.47 (0.54-3.97) | 0.45 |
| **Gastrointestinal Outcomes** | | | | | | |
| **Gastrointestinal Ulcer** | 3 [18,22-24] | 0.89 | 0% | Fixed | RR 0.36 (0.18-0.71) | 0.003 |
| **Gastrointestinal Bleeding** | 8 [17-25] | ＜0.00001 | 0% | Fixed | RR 0.31 (0.22-0.44) | 0.004 |
| **Upper Gastrointestinal Bleeding** | 4 [17,19,22-24] | 0.38 | 2% | Fixed | RR 0.35 (0.20-0.62) | 0.003 |

CI, confidence interval; PPI, proton pump inhibitor; RR, risk ratio
